# Research on action behavior of neuron system in case of single pulse stimulus

**DOI:** 10.1038/s41598-020-58100-9

**Published:** 2020-01-27

**Authors:** Mingliang Zhang, Menghua Man, Guilei Ma, Meiyu Ye, Shanghe Liu

**Affiliations:** 1 National Key Laboratory on Electromagnetic Environment Effects, National Key Laboratory on Electromagnetic Environment Effects, Shijiazhuang, Hebei 050003 China; 2 School of Mechanical Engineering, Shijiazhuang Tiedao University, Shijiazhuang, Hebei 050043 China; 3 Department of Foreign Language, Shijiazhuang Tiedao University, Shijiazhuang, Hebei 050043 China

**Keywords:** Voltage clamp, Dynamical systems

## Abstract

Facing on the complex electromagnetic environment of electrical equipment, based on the bio-anti-interference characteristics of neuron system, the bio-inspired electromagnetic protection is proposed in order to improve and assist the traditional electromagnetic protection method. In order to analyze the dynamical characteristics of electrical signal transfer process of neuron system, Hodgkin-Huxley (HH) model is adopted to calculate the action potential of single neuron. The initial value problem used in the parameters of Hodgkin-Huxley model is studied in order to satisfy the physiological phenomenon. The stability of HH model is analyzed to assess the dynamic stable performance of neuron. Based on the investigation of single neuron, a simple neuron system consisted of two neurons and one synapse is studied. The compassion between the action potential of posterior neuron and different synapse is performed, which explores how the mathematic models of different synapses influence the action potential. The relationship between action potential of posterior neuron and coupling strength of simplified synapse is calculated to explain the diversity of electrical signal output of neuron system. These numerical results enable to provide some datum for deeply developing the bio-inspired electromagnetic protection and well designing the bio-inspired circuit.

## Introduction

As the fast development and wide application of integrated circuit, the problem of electromagnetic protection for electrical equipment becomes more and more difficult. The failure of electrical equipment due to the strong electromagnetic interference will cause the key machine and weapon to seriously damage. Furthermore, the modern electrical equipment needs not only the higher electromagnetic sensibility but also more strong electromagnetic anti-interference performance, which proposes the large challenge for the electromagnetic protection. The traditional electromagnetic protection measures, such as ground connection, shield, isolation, smoothing, is adopted to resist the complex electromagnetic environment, which has obtained a series of achievements. However the normal working state of electrical equipment is hard to be kept by way of single measure and their combined measures. Except from improving the performance of traditional electromagnetic protection method, based on the bio-anti-interference phenomenon and behavior, the bio-inspired electromagnetic protection is proposed^1^, which provides a novel concept for electromagnetic protection. Deeply studying the bio-inspired electromagnetic protection method can enrich the form of electromagnetic protection, which can assist and compensate the traditional method in order to obtain the better electromagnetic protection effect. Thus, the bio-inspired electromagnetic protection has the enormous meaning. The foundation of bio-inspired electromagnetic protection is mainly dependent on the performance of bio-anti-interference and its final goal is to design the bio-inspired circuit for improving the working performance of the electrical equipment even in case of complex electromagnetic environment.

Nowadays, the bio-inspired electromagnetic protection is mainly focused on the some biological mechanism. The neuron system of human still performs the sensible action behavior even in the complex electromagnetic environment, which is a typical example as bio-anti-interference against electromagnetic environment. Meanwhile, it can handle the complex signal, dominate the sapiential physiology behavior, memory, learn and think due to the physiology structure and unit^2^. The neuron system of human includes the abundant input-output characteristics for dealing with the complex information, thus the neuron system is able to be used in the bio-inspired electromagnetic protection, which needs to be widely investigated.

A simple neuron system consisted of two neurons and one synapse is mainly studied in this manuscript. The dynamic characteristics of neuron in case of electromagnetic interfere can provide an important theoretical foundation for bio-inspired electromagnetic protection, the analysis of the dynamic characteristics is performed by the mathematic model, thus the mathematic model of neuron and influence of electromagnetic field versus neuron is summarized. Hodgkin and Huxley propose the famous Hodgkin-Huxley model by way of experiment, which is the most close to the actual biological mechanism. They systemically detail the membrane current, membrane potential (action potential), membrane conductivity and activation probability and deactivation probability of some ion passage^3–6^. Thus, Hodgkin-Huxley model plays an important role in a variety of the mathematic model of neuron. In order to simplify the mathematical model of Hodgkin-Huxley model, some other models of neuron, such as FHN, ML, HR, are proposed^7–10^. Based on the model of neuron, Deng studies the problem of chaos and synchronization in case of external electrical field^11^ and Lv use the memristor to study the dynamic characteristics of neuron in case of electromagnetic radiation^12,13^. Based on the analysis of single neuron, the connection section (synapse) between two neurons can influence the transfer process of electrical signal, which pays some attention of many researchers. In order to investigate the relationship between input and output, some mathematic models of synapse are proposed. Kandle proposes a model of dynamic chemical synapse^14^, Hansel proposes a kind of the simplest mathematic model of chemical synapse^15^, Destexhe proposes a kind of dynamic model of the transfer process of chemical synapse^16^. Based on the transfer of neurotransmitter, Sharp proposes a kind of the mathematic model of chemical synapse^17,18^. Wang studies the synapse suppression network of hippocampal neuron to propose a model of chemical synapse^19^. Rabinovich establishes a model of chemical synapse with the delay hysteresis^20^. Savtchenko proposes a mathematic model of chemical synapse based on the complex process of synapse transfer^21^. Chen, Lu and Wen compare the performance of several chemical synapses by way of numerically analyzing the output electrical signal^22^. Except from the chemical synapse, two neurons can also be connected with the electrical synapse.

Based on the above analysis, although the bio-inspired electromagnetic protection develops in a short time, some achievements have been obtained. Moreover, the systematic investigation is eager to be performed. This manuscript mainly investigates the dynamic characteristics of neuron system and acquires some numerical results, which provides some datum and parameters for the bio-inspired electromagnetic protection. The initial value problem of Hodgkin-Huxley model is studied in order to obtain the satisfied biological action potential, which is validated by way of simulation. The stability problem of HH equation is analyzed, which can estimate the performance of neuron. The action potential of posterior neuron is compared between the different types of synapses. The simplified synapse connected with two neurons is mainly focused on and the relationship between action potential of posterior neuron and the coupling strength is analyzed.

## The Components of Neuron System and Electrical Signal Transfer

**Figure 1:**
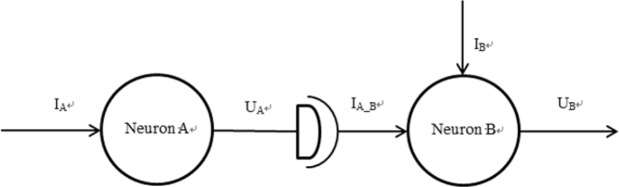
The equivalent electrical signal transfer of simple neuron system.

The most simple neuron system is assumed to be consisted of two neurons and one connection section (synapse). In order to easily describe the function of two neurons, the preceding neuron is named as neuron A, while the posterior neuron is named as neuron B. The electrical signal transfer process of simple neuron system is assumed to only perform from neuron A to neuron B, as shown in Fig. 1.

The stimuli current A (I_A_) is exerted to neuron A, while stimuli current B (I_B_) is exerted to neuron B. The whole working principle of electrical signal transfer is as follow:

- The stimuli current A stimulates neuron A, and then the neuron A creates a series of complex action processes, the action potential deliver toward the section of axon.
- The section of axon creates a series of complicated action processes, the action potential of preceding neuron exchanges into the transfer current I_A_B_, which can affect the dendrite of neuron B.
- The stimuli current I_B_ and transfer current I_A_B_ cooperatively provoke the neuron B to create the action potential.

Based on three steps, it is clearly seen that the action potential of neuron B is influenced not only self-stimuli current I_B_ but also transfer current I_A_B_ by way of synapse. The action potential of neuron B is mainly focused on.

## The Initial Value Problem of Single Neuron and Analysis

### Hodgkin-Huxley model

**Figure 2:**
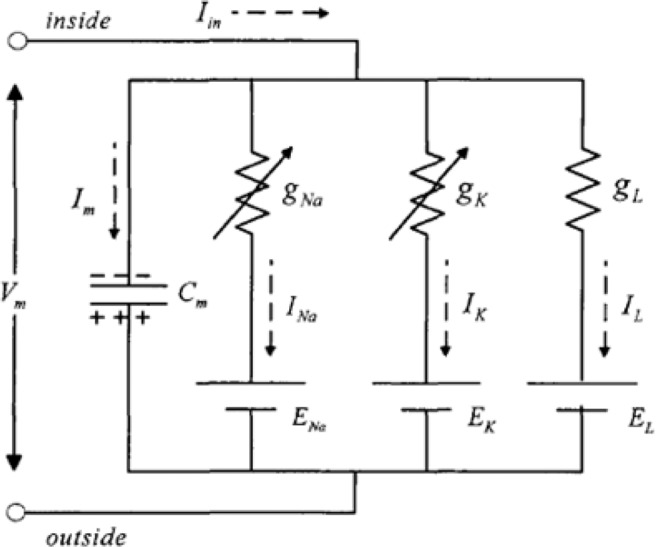
The equivalent circuit of Hodgkin-Huxley model.

Hodgkin-Huxley model^3–6^ is an efficient mathematic tool for analyzing the dynamic characteristics of neuron, which has the definite biological meaning, such as membrane potential, sodium ion passage, potassium ion passage. The equivalent circuit of Hodgkin-Huxley model is shown in Fig. 2.

Based on the circuit theory, the differential equation of Hodgkin-Huxley model is expressed as

1 $$\left\{\begin{array}{c}{{\rm{C}}}_{1}\frac{d{V}_{1}}{dt}={{\rm{gNa}}}_{1}({{\rm{E}}}_{{\rm{Na}}1}-{{\rm{V}}}_{1})+{{\rm{gK}}}_{1}({{\rm{E}}}_{{{\rm{K}}}_{1}}-{{\rm{V}}}_{1})+{{\rm{G}}}_{L1}({{\rm{E}}}_{{\rm{L}}1}-{{\rm{V}}}_{1})+{{\rm{I}}}_{1}\\ {{\rm{gNa}}}_{1}={{\rm{G}}}_{{\rm{Na}}1}{{{\rm{m}}}_{1}}^{3}{{\rm{h}}}_{1}\\ {{\rm{gK}}}_{1}={{\rm{G}}}_{{\rm{K}}1}{{{\rm{n}}}_{1}}^{4}\\ \frac{{{\rm{dm}}}_{1}}{{\rm{dt}}}={\alpha }_{{\rm{m}}1}(1-{{\rm{m}}}_{1})-{\beta }_{{\rm{m}}1}{{\rm{m}}}_{1}\\ \frac{{{\rm{dh}}}_{1}}{{\rm{dt}}}={\alpha }_{{\rm{h}}1}(1-{{\rm{h}}}_{1})-{\beta }_{{\rm{h}}1}{{\rm{h}}}_{1}\\ \frac{{{\rm{dn}}}_{1}}{{\rm{dt}}}={\alpha }_{{\rm{n}}1}(1-{{\rm{n}}}_{1})-{\beta }_{{\rm{n}}1}{{\rm{n}}}_{1}\\ {\alpha }_{{\rm{m}}1}=\frac{0.1({{\rm{V}}}_{1}-{{\rm{V}}}_{{\rm{rest}}1}-25)}{1-{{\rm{e}}}^{-({{\rm{V}}}_{1}-{{\rm{V}}}_{{\rm{rest}}1}-25)/10}}\\ {\beta }_{{\rm{m}}1}=4{{\rm{e}}}^{-({{\rm{V}}}_{1}-{{\rm{V}}}_{{\rm{rest}}1})/18}\\ {\alpha }_{{\rm{h}}1}=0.07{{\rm{e}}}^{-({{\rm{V}}}_{1}-{{\rm{V}}}_{{\rm{rest}}1})/20}\\ {\beta }_{{\rm{h}}1}=\frac{1}{1+{{\rm{e}}}^{-({{\rm{V}}}_{1}-{{\rm{V}}}_{{\rm{rest}}1}-30)/10}}\\ {\alpha }_{{\rm{n}}1}=\frac{0.01({{\rm{V}}}_{1}-{{\rm{V}}}_{{\rm{rest}}1}-10)}{1-{{\rm{e}}}^{-({{\rm{V}}}_{1}-{{\rm{V}}}_{{\rm{rest}}1}-10)/10}}\\ {\beta }_{n1}=0.125{e}^{-({{\rm{V}}}_{1}-{{\rm{V}}}_{{\rm{rest}}1})/80}\end{array}\right.$$

Where C_1_ indicates the membrane capacitance of single neuron, V_1_ indicates the action potential of single neuron, gNa_1_ indicates the sodium conductance of single neuron, gK_1_ indicates the potassium conductance of single neuron, G_Na1_ indicates the maximum sodium conductance of single neuron, G_K1_ indicates the maximum potassium conductance of single neuron, G_L1_ indicates the maximum leak conductance of single neuron, E_Na1_ indicates the reversal sodium potential of single neuron, E_K1_ indicates the reversal potassium potential of single neuron, E_L1_ indicates the reversal leak potential of single neuron, m_1_ indicates the activation parameter of sodium ion of single neuron, h_1_ indicates the deactivation parameter of sodium ion of single neuron, n_1_ indicates the activation parameter of potassium ion of single neuron, I_1_ indicates the stimuli current of single neuron, V_rest1_ indicates the rest potential of single neuron.

It is easily observed that the Eq. (1) has strong nonlinear and coupling characteristics, which brings us some difficulties to solve the solution analytically. The numerical calculation provides an effective method to calculate the differential equation in case of initial condition, which is adopted in our manuscript. In the numerical calculation, the initial condition (initial value) is necessary and important because the abnormal behavior happens in case of inappropriate initial value due to the strong nonlinear and coupling characteristics of Eq. (1). According to the Eq. (1) of Hodgkin-Huxley model, when the initial value is not appropriate, some special abnormal phenomenon of action potential, such as slow increase, slow decrease, two spikes, are observed, which is bad in agreement with the normal biological behavior seen in next section 3.3. Therefore, the initial value problem needs to be studied in this section.

### The initial value of biological parameters and analysis

In order to calculate the equation (1), V_1_, m_1_ h_1_ and n_1_ need to be given as the initial value (V_01_, m_01_, h01, n_01_). Based on the ideal biological behavior, the action potential of single neuron creates a spike until the stimuli current exerts on the neuron. The stimuli beginning time of stimuli current is defined as t_sti1_(t_sti_ > 0). In the initial state (t = 0 ms), the initial stimuli current I_01_ is equal to 0 and the initial action potential V_01_ is equal to V_rest1_ (V_01_ = V_rest1_). Furthermore, when the time t is less than t_sti1_, the Eq. (1) should be first met as

2 $${G}_{Na1}{m}_{1}{(t)}^{3}h(t)({E}_{Na1}-{V}_{01})+{G}_{K1}{n}_{1}{(t)}^{4}({E}_{K1}-{V}_{01})+{G}_{L1}({E}_{L1}-{V}_{01})+0={C}_{1}\frac{d{V}_{1}(t)}{dt}=0$$

Where 0 in the left of Eq. (2) indicates that the stimuli current is equal to 0 (t < t_sti1_), dV_1_(t)/dt (t < t_sti1_) is equal to 0, which implies that the action potential keeps fixed until the stimuli current activates.

Then

3 $${G}_{Na1}{m}_{1}{(t)}^{3}{h}_{1}(t)({E}_{Na1}-{V}_{01})+{G}_{K1}{n}_{1}{(t)}^{4}({E}_{K1}-{V}_{01})+{G}_{L1}({E}_{L1}-{V}_{01})=0$$

If the Eq. (4) is satisfied, the following conditions should be met as

4 $$\left\{\begin{array}{c}{G}_{Na1}{{m}_{01}}^{3}{h}_{01}({E}_{Na1}-{V}_{01})+{G}_{K1}{{n}_{01}}^{4}({E}_{K1}-{V}_{01})+{G}_{L1}({E}_{L1}-{V}_{01})=0\\ {\alpha }_{m01}(1-{m}_{01})-{\beta }_{m01}{m}_{01}=\frac{d{m}_{1}(t)}{dt}=0\\ {\alpha }_{h01}(1-{h}_{01})-{\beta }_{h01}{h}_{01}=\frac{d{h}_{1}(t)}{dt}=0\\ {\alpha }_{n01}(1-{n}_{01})-{\beta }_{n01}{n}_{01}=\frac{d{n}_{1}(t)}{dt}=0\end{array}\right.$$

Then

5 $$\left\{\begin{array}{c}{V}_{01}=\frac{{G}_{Na1}{{m}_{01}}^{3}{h}_{01}{E}_{Na1}+{G}_{K1}{{n}_{01}}^{4}{E}_{K1}+{G}_{L1}{E}_{L1}}{{G}_{Na1}{{m}_{01}}^{3}{h}_{01}+{G}_{K1}{{n}_{01}}^{4}+{G}_{L1}}\\ {m}_{01}=\frac{{\alpha }_{m01}({V}_{01})}{{\alpha }_{m01}({V}_{01})+{\beta }_{m01}({V}_{01})}\\ {h}_{01}=\frac{{\alpha }_{h01}({V}_{01})}{{\alpha }_{h01}({V}_{01})+{\beta }_{h01}({V}_{01})}\\ {n}_{01}=\frac{{\alpha }_{n01}({V}_{01})}{{\alpha }_{n01}({V}_{01})+{\beta }_{n01}({V}_{01})}\end{array}\right.$$

Based on the Eq. (1), the ion coefficients are readily gained as

6 $$\left\{\begin{array}{c}{\alpha }_{m01}({V}_{01})=-\frac{2.5}{1-{e}^{25/10}}\\ {\beta }_{m01}({V}_{01})=4{e}^{-(0)/18}=4\\ {\alpha }_{h01}({V}_{01})=0.07{e}^{-(0)/20}=0.07\\ {\beta }_{h01}({V}_{01})=\frac{1}{1+{e}^{3}}\\ {\alpha }_{n01}({V}_{01})=\frac{-0.1}{1-e}\\ {\beta }_{n01}({V}_{01})=0.125{e}^{-(0)/80}=0.125\end{array}\right.$$

Based on the Eqs. (5) and (6), the initial values of parameters are readily calculated: m_01_ = 0.0529, h_01_ = 0.5961, n_01_ = 0.3177. In order to calculate the initial action potential V_01_, a series of parameters need to be given, as list in Table 1.

**Table 1:** The biological parameters of neuron.

Parameter	Value
G_Na1_	120 mS/cm^2^
G_K1_	36 mS/cm^2^
G_L1_	0.3 mS/cm^2^
E_Na1_	50 mV
E_K1_	−77 mV
E_L1_	−54.4 mV

Based on the value of biological parameters in Table 1, the parameter V_01_ is calculated to be −64.9995 mV, which is good in agreement with the experimental value (−65 mV). It implies that the initial values of biological parameters in this section are validated.

#### The relationship between action behavior of single neuron and initial value of biological parameters and analysis

Due to the nonlinear and coupling characteristics of Eq. (1), it is hard to solve the Eq. (1) analytically. 4-order Runge-Kutta algorithm, an efficient numerical algorithm with high calculated precision, is adopted to calculate the Eq. (1), which gives us some useful consequences. When the parameters of V_0_, m_0_, h_0_, n_0_, get the different initial value, the gate coefficient and conductance of sodium and conductance of potassium and action potential (action behavior) are different. The simulated parameters are: the membrane capacitance C is equal to 1 μF/cm^2^, the stimuli current intensity is a single pulse signal: 100 μA/cm^2^ amplitude and 2 ms pulse width. In order to study the influence between the initial value and action behavior, the typical simulated parameters are list in Table 2.

**Table 2:** The initial value of parameters of three cases.

	V_01_	m_01_	h_01_	n_01_
Case 1	−65 mV	0	0	0
Case 2	−65 mV	0.0529	0.5961	0.3177
Case 3	−65 mV	0.1	0.7	0.4

**Figure 3:**
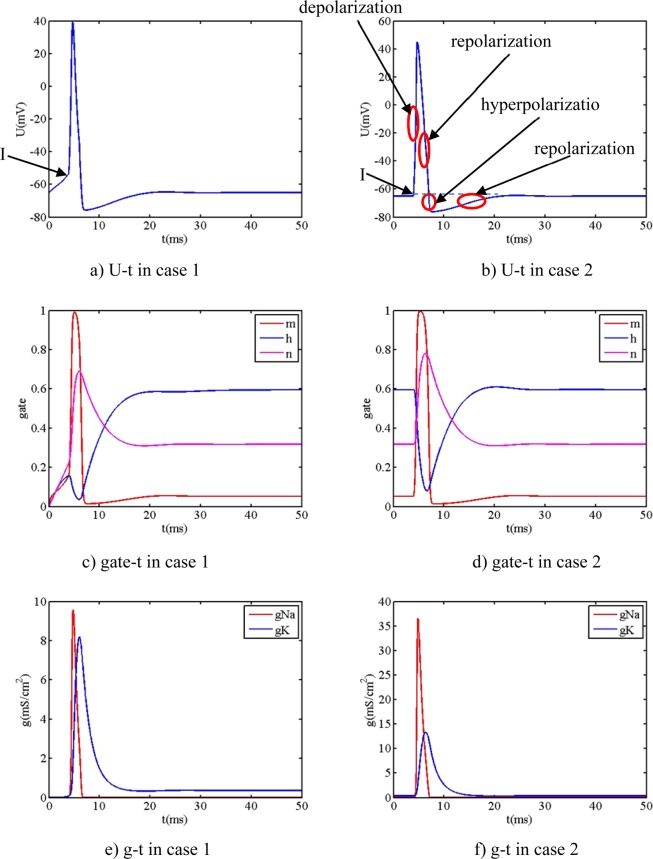
t_sti1_ = 4 ms the action potential and gate coefficient and ion conductance.

**Figure 4:**
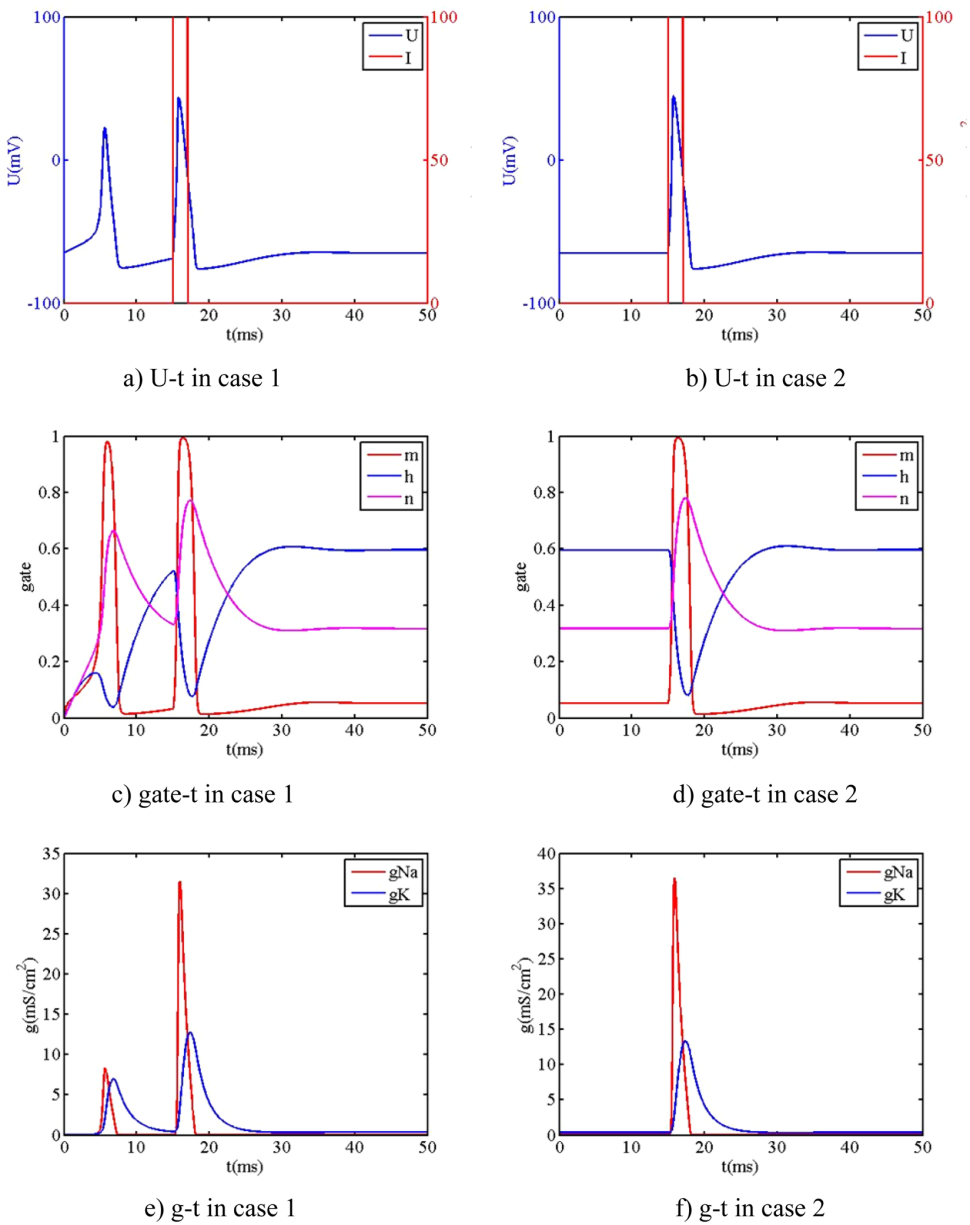
t_sti1_ = 15 ms the action potential and gate coefficient and ion conductance.

**Figure 5:**
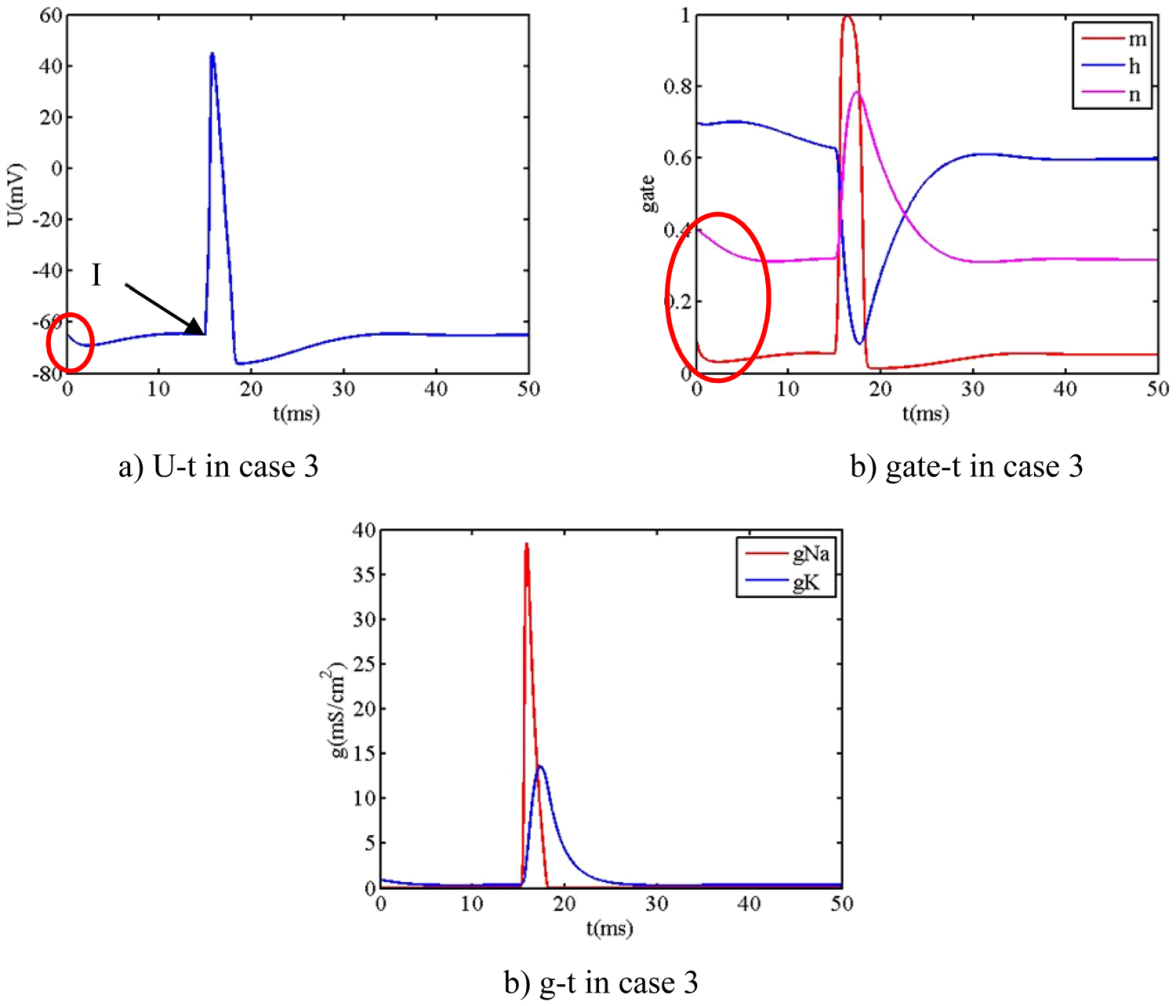
t_sti1_ = 15 ms the action potential and gate coefficient and ion conductance.

It is readily observed that the parameters of case 2 in Table 2 are the same to the value obtained in section 3.2. The values of other simulated parameters are obtained from Table 1 in section 3.2. The relationship between action behavior and initial value are obtained, as shown in Figs. 3–5.

From Fig. 3, four consequences are readily observed: (1) The action potential (figure a) initially slowly increases before the stimuli current activates, then dramatically increases when the stimuli current begins to activate, then dramatically decreases then slowly increases then stabilizes at the rest potential V_rest1_ as time passes. The action potential (figure b) keeps fixed before the stimuli current activates, then dramatically increases when the stimuli current begins to activate, then dramatically decreases then slowly increases then stabilizes at the rest potential V_rest1_ as time passes, which reflects the biological whole process of depolarization, repolarization, hyperpolarization and repolarization. (2) The gate m response (figure c) slowly increases before the stimuli current activates then dramatically increases when the stimuli current begins to activate, then dramatically decreases then slowly increases then stabilizes at the initial value m_01_ as time passes, whose varied regularity is similar to the one of action potential (figure a). The gate m response (figure d) keeps fixed before the stimuli current activates, then dramatically increases when the stimuli current begins to activate, then dramatically decreases then slowly increases then stabilizes at the initial value m_01_ as time passes, whose varied regularity is similar to the one of action potential (figure b). The varied behavior of n gate is similar to the one of m gate. (3) The varied behavior of h gate is large different from the one of m gate. The gate h response (figure c) initially increases to extreme then decreases then increase then stabilizes as time passes, while the gate h response (figure d) keeps fixed then decreases then increases then stabilizes as time passes. (4) The ion conductance of sodium and potassium (figure e and f) has the similar varied behavior that they both initially increase then decrease as time passes, but they has some remarkable difference that the maximum in case 1 are 9.6 mS/cm^2^ and 8.2 mS/cm^2^ respectively, while the maximum in case 2 are 36.5 mS/cm^2^ and 13.3 mS/cm^2^ respectively.

From Fig. 4, if gate m_01_, h_01_, n_01_ adopt the different value, some more special obvious phenomena are observed when the stimuli current begins to activate at 15 ms: (1) The action potential spike (figure a) begins to happen before the stimuli current begins to activate, which implies that the initial biological electrical state of neuron model is not stable (not at the resting state) if the initial value of neuron model is not appropriate. Then the action potential again begins to quickly vary when the stimuli current begins to activate. (2) The amplitude of first spike (about 20 mV) is less than the one of second spike (about 44 mV). (3) The corresponding gate coefficients m, n and h (figure c) occur to have two spikes, which is of large difference from the gate m, n and h (figure d) that neuron model adopts a set of appropriate value. (4) The ion conductance of sodium and potassium (figure e) has the similar varied behavior that two spikes both initially increase then decrease as time passes, moreover, two spikes has remarkable difference that the maximum of first peak are 8.3 mS/cm^2^ and 7.0 mS/cm^2^ respectively while the maximum of second peak are 31.5 mS/cm^2^ and 12.8 mS/cm^2^, respectively, which are both different from the ones neuron model adopts a set of appropriate value (36.5 mS/cm^2^ and 13.3 mS/cm^2^).

From Fig. 5, when the initial value m_0_ = 0.1, h_0_ = 0.7, n_0_ = 0.4 of neuron model is adopted, it is observed that the action potential and the gate n and h and ion conductance all initially decrease.

From above three set of simulated parameters, the action potential and gate coefficients and ion conductance are all influenced by the time of stimuli current and the initial value of gate m_1_, n_1_ and h_1_. Especially in case of the gate m_01_, h_01_, n_01_ is not appropriate, some special behavior are observed: (1) Two spikes of action potential can be observed. (2) The initial slow increase and initial slow decrease of the action potential can be observed. The detailed influence between the action behavior and simulated parameters (initial value of model and value of biological parameters and stimuli time and the amplitude and lasting time of stimuli current) is beyond the scope of our manuscript, which is able to need the bifurcation and chaos theory due to the nonlinear characteristics of neuron model.

## The Stability Problem of Single Neuron and Analysis

The working stability of neuron is of importance for the foundation of bio-inspired electromagnetic protection, which is investigated by way Lyapunov stability theory (indirect method) in this section. In order to analyze the stability of neuron, HH model is adopted. To simplify the analysis, the autonomous system (the mathematic equation of HH model) is only studied in our manuscript. Thus, the non-autonomous system (HH equation) needs to transform into autonomous system (transforming HH equation), then the stimuli current I_1_(t) should be equal to 0. The transforming HH equation is expressed as

7 $$\left\{\begin{array}{c}\frac{{{\rm{dV}}}_{1}}{{\rm{dt}}}=\frac{{{\rm{gNa}}}_{1}}{{{\rm{C}}}_{1}}({{\rm{E}}}_{{\rm{Na}}1}-{{\rm{V}}}_{1})+\frac{{{\rm{gK}}}_{1}}{{{\rm{C}}}_{1}}({E}_{{K}_{1}}-{V}_{1})+\frac{{G}_{L1}}{{{\rm{C}}}_{1}}({E}_{L1}-{V}_{1})\\ {{\rm{gNa}}}_{1}={{\rm{G}}}_{{\rm{Na}}1}{{{\rm{m}}}_{1}}^{3}{{\rm{h}}}_{1}\\ {{\rm{gK}}}_{1}={{\rm{G}}}_{{\rm{K}}1}{{{\rm{n}}}_{1}}^{4}\\ \frac{{{\rm{dm}}}_{1}}{{\rm{dt}}}={\alpha }_{{\rm{m}}1}(1-{{\rm{m}}}_{1})-{\beta }_{{\rm{m}}1}{{\rm{m}}}_{1}\\ \frac{{{\rm{dh}}}_{1}}{{\rm{dt}}}={\alpha }_{{\rm{h}}1}(1-{{\rm{h}}}_{1})-{\beta }_{{\rm{h}}1}{{\rm{h}}}_{1}\\ \frac{{{\rm{dn}}}_{1}}{{\rm{dt}}}={\alpha }_{{\rm{n}}1}(1-{{\rm{n}}}_{1})-{\beta }_{{\rm{n}}1}{{\rm{n}}}_{1}\\ {\alpha }_{{\rm{m}}1}=\frac{0.1({{\rm{V}}}_{1}-{{\rm{V}}}_{{\rm{rest}}1}-25)}{1-{{\rm{e}}}^{-({{\rm{V}}}_{1}-{{\rm{V}}}_{{\rm{rest}}1}-25)/10}}\\ {\beta }_{{\rm{m}}1}=4{{\rm{e}}}^{-({{\rm{V}}}_{1}-{{\rm{V}}}_{{\rm{rest}}1})/18}\\ {\alpha }_{{\rm{h}}1}=0.07{{\rm{e}}}^{-({{\rm{V}}}_{1}-{{\rm{V}}}_{{\rm{rest}}1})/20}\\ {\beta }_{{\rm{h}}1}=\frac{1}{1+{{\rm{e}}}^{-({{\rm{V}}}_{1}-{{\rm{V}}}_{{\rm{rest}}1}-30)/10}}\\ {\alpha }_{n1}=\frac{0.01({{\rm{V}}}_{1}-{{\rm{V}}}_{{\rm{rest}}1}-10)}{1-{e}^{-({{\rm{V}}}_{1}-{{\rm{V}}}_{{\rm{rest}}1}-10)/10}}\\ {\beta }_{n1}=0.125{{\rm{e}}}^{-({{\rm{V}}}_{1}-{{\rm{V}}}_{{\rm{rest}}1})/80}\end{array}\right.$$

The working equilibrium position (point) needs to be sought in order to perform the next coordinate transformation. The equilibrium point has been solved by the initial value problem of single neuron in section 3.2.

Oder

$${\boldsymbol{u}}=\left(\begin{array}{c}{{u}}_{{1}}\\ {{u}}_{{2}}\\ {{u}}_{{3}}\\ {{u}}_{{4}}\end{array}\right)=\left(\begin{array}{c}{V}\\ {m}\\ {h}\\ {n}\end{array}\right)$$

Then, the equilibrium point **u**_**s**_ is expressed as

$${{\boldsymbol{u}}}_{{\boldsymbol{s}}}=\left(\begin{array}{c}{{V}}_{{s}}\\ {{m}}_{{s}}\\ {{h}}_{{s}}\\ {{n}}_{{s}}\end{array}\right)=\left(\begin{array}{c}{{V}}_{{01}}\\ {{m}}_{{01}}\\ {{h}}_{{01}}\\ {{n}}_{{01}}\end{array}\right)$$

Order

$${\boldsymbol{v}}=\left(\begin{array}{c}{{v}}_{{1}}\\ {{v}}_{{2}}\\ {{v}}_{{3}}\\ {{v}}_{{4}}\end{array}\right)$$

$${\boldsymbol{u}}=\left(\begin{array}{c}{{u}}_{{1}}\\ {{u}}_{{2}}\\ {{u}}_{{3}}\\ {{u}}_{{4}}\end{array}\right)=\left(\begin{array}{c}{{v}}_{1}+{{V}}_{01}\\ {{v}}_{2}+{{m}}_{01}\\ {{v}}_{3}+{{h}}_{01}\\ {{v}}_{4}+{{n}}_{01}\end{array}\right)$$

$${\rm{When}}\,{{\boldsymbol{v}}}_{s}=\left(\begin{array}{c}{{v}}_{1s}\\ {{v}}_{2{s}}\\ {{v}}_{3{s}}\\ {{v}}_{4{s}}\end{array}\right)=\left(\begin{array}{c}0\\ 0\\ 0\\ 0\end{array}\right){\rm{then}}\,{{\boldsymbol{u}}}_{s}=\left(\begin{array}{c}{{V}}_{01}\\ {{m}}_{01}\\ {{h}}_{01}\\ {{n}}_{01}\end{array}\right)$$

and

$$\mathop{{\boldsymbol{u}}}\limits^{g}=\left(\begin{array}{c}\mathop{{{u}}_{{1}}}\limits^{g}\\ \mathop{{{u}}_{{2}}}\limits^{g}\\ \mathop{{{u}}_{{3}}}\limits^{g}\\ \mathop{{{u}}_{{4}}}\limits^{g}\end{array}\right)=\left(\begin{array}{c}\mathop{{{v}}_{{1}}}\limits^{g}\\ \mathop{{{v}}_{{2}}}\limits^{g}\\ \mathop{{{v}}_{{3}}}\limits^{g}\\ \mathop{{{v}}_{{4}}}\limits^{g}\end{array}\right)=\mathop{{\boldsymbol{v}}}\limits^{g}$$

The initial HH equation is expressed by way of ***v*** as

8 $$\left\{\begin{array}{c}\begin{array}{c}\frac{{{\rm{dv}}}_{1}}{{\rm{dt}}}=\frac{{{\rm{G}}}_{{\rm{Na}}1}{({{\rm{v}}}_{2}+{{\rm{m}}}_{01})}^{3}({{\rm{v}}}_{3}+{{\rm{h}}}_{01})}{{{\rm{C}}}_{1}}({{\rm{E}}}_{{\rm{Na}}1}-{{\rm{v}}}_{1}-{{\rm{V}}}_{01})+\frac{{{\rm{G}}}_{{\rm{K}}1}{({{\rm{v}}}_{4}+{{\rm{n}}}_{01})}^{4}}{{{\rm{C}}}_{1}}({{\rm{E}}}_{{\rm{K}}1}-{{\rm{v}}}_{1}-{{\rm{V}}}_{01})+\frac{{{\rm{G}}}_{{\rm{L}}1}}{{{\rm{C}}}_{1}}({{\rm{E}}}_{{\rm{L}}1}-{{\rm{v}}}_{1}-{{\rm{V}}}_{01})\end{array}\\ \frac{{{\rm{dv}}}_{2}}{{\rm{dt}}}=\frac{0.1({{\rm{v}}}_{1}-25)}{1-{e}^{-({{\rm{v}}}_{1}-25)/10}}(1-{{\rm{v}}}_{2}-{{\rm{m}}}_{01})-4{{\rm{e}}}^{-{v}_{1}/18}({{\rm{v}}}_{2}+{{\rm{m}}}_{01})\\ \frac{{{\rm{dv}}}_{3}}{{\rm{dt}}}=0.07{{\rm{e}}}^{-{{\rm{v}}}_{1}/20}(1-{{\rm{v}}}_{3}-{{\rm{h}}}_{01})-\frac{1}{1+{{\rm{e}}}^{-({{\rm{v}}}_{1}-30)/10}}({{\rm{v}}}_{3}+{{\rm{h}}}_{01})\\ \frac{{{\rm{dv}}}_{4}}{{\rm{dt}}}=\frac{0.01({v}_{1}-10)}{1-{{\rm{e}}}^{-({{\rm{v}}}_{1}-10)/10}}(1-{{\rm{v}}}_{4}-{{\rm{n}}}_{01})-0.125{{\rm{e}}}^{-{{\rm{v}}}_{1}/80}({{\rm{v}}}_{4}+{{\rm{n}}}_{01})\end{array}\right.$$

Based on Lyapunov stability theory (indirect method), the derived system first needs to be deduced. At the equilibrium point (0, 0, 0), the derived linear equation of HH model is expressed as based on Taylor expansion

9 $$\left(\begin{array}{c}\mathop{{{v}}_{1}}\limits^{g}\\ \mathop{{{v}}_{2}}\limits^{g}\\ \mathop{{{v}}_{3}}\limits^{g}\\ \mathop{{{v}}_{4}}\limits^{g}\end{array}\right)=\left(\begin{array}{c}\begin{array}{c}{{v}}_{1}\left(-\frac{{{\rm{G}}}_{{\rm{N}}{\rm{a}}1}}{{{\rm{C}}}_{1}}{{{\rm{m}}}_{01}}^{3}{{\rm{h}}}_{01}-\frac{{{\rm{G}}}_{{\rm{K}}1}}{{{\rm{C}}}_{1}}{{{\rm{n}}}_{01}}^{4}-\frac{{{\rm{G}}}_{{\rm{L}}1}}{{{\rm{C}}}_{1}}\right)+{{v}}_{2}\frac{3{{\rm{G}}}_{{\rm{N}}{\rm{a}}1}}{{{\rm{C}}}_{1}}{{{\rm{m}}}_{01}}^{2}{{\rm{h}}}_{01}\left({{\rm{E}}}_{{\rm{N}}{\rm{a}}1}-{V}_{01}\right)\\ +{{v}}_{3}\frac{{{\rm{G}}}_{{\rm{N}}{\rm{a}}1}}{{{\rm{C}}}_{1}}{{{\rm{m}}}_{01}}^{3}\left({{\rm{E}}}_{{\rm{N}}{\rm{a}}1}-{{\rm{V}}}_{01}\right)+{{v}}_{4}\frac{4{{\rm{G}}}_{K}}{{{\rm{C}}}_{1}}{{{\rm{n}}}_{01}}^{3}\left({{\rm{E}}}_{{\rm{K}}1}-{{\rm{V}}}_{01}\right)\end{array}\\ {v}_{1}\left(\frac{\left(1-{{\rm{m}}}_{01}\right)\left(0.1+0.15{{\rm{e}}}^{2.5}\right)}{{\left(1-{e}^{2.5}\right)}^{2}}+\frac{2}{9}{{\rm{m}}}_{01}\right)+{{\rm{v}}}_{2}\left(-4+\frac{2.5}{1-{{\rm{e}}}^{2.5}}\right)\\ {v}_{1}\left(-\frac{7}{2000}\left(1-{{\rm{h}}}_{01}\right)-\frac{{{\rm{e}}}^{3}}{10{\left(1+{{\rm{e}}}^{3}\right)}^{2}}{{\rm{h}}}_{01}\right)+{{\rm{v}}}_{3}\left(-0.07-\frac{1}{1+{{\rm{e}}}^{3}}\right)\\ {{\rm{v}}}_{1}\left(\frac{0.01\left(1-{{\rm{n}}}_{01}\right)}{{\left(1-{\rm{e}}\right)}^{2}}+\frac{1}{640}{{\rm{n}}}_{01}\right)+{{\rm{v}}}_{4}\left(-\frac{1}{8}+\frac{0.1}{1-{\rm{e}}}\right)\end{array}\right)$$

The Eq. (9) is expressed as the state equation

10 $$\mathop{{\boldsymbol{v}}}\limits^{g}=A{\boldsymbol{v}}$$

Where A indicates Jacobian matrix

$${\rm{A}}=\left(\begin{array}{cccc}-\frac{{{\rm{G}}}_{Na1}}{{{\rm{C}}}_{1}}{{{\rm{m}}}_{01}}^{3}{{\rm{h}}}_{01}-\frac{{{\rm{G}}}_{K1}}{{{\rm{C}}}_{1}}{{{\rm{n}}}_{01}}^{4}-\frac{{{\rm{G}}}_{L1}}{{{\rm{C}}}_{1}} & \frac{3{{\rm{G}}}_{Na1}}{{{\rm{C}}}_{1}}{{{\rm{m}}}_{01}}^{2}{{\rm{h}}}_{01}({{\rm{E}}}_{Na1}-{{\rm{V}}}_{01}) & \frac{{{\rm{G}}}_{Na1}}{{{\rm{C}}}_{1}}{{{\rm{m}}}_{01}}^{3}({{\rm{E}}}_{Na1}-{{\rm{V}}}_{01}) & \frac{4{{\rm{G}}}_{{\rm{K}}}}{{{\rm{C}}}_{1}}{{{\rm{n}}}_{01}}^{3}({{\rm{E}}}_{K1}-{{\rm{V}}}_{01})\\ \frac{(1-{{\rm{m}}}_{01})(0.1+0.15{{\rm{e}}}^{2.5})}{{(1-{{\rm{e}}}^{2.5})}^{2}}+\frac{2}{9}{{\rm{m}}}_{01} & -4+\frac{2.5}{1-{e}^{2.5}} & 0 & 0\\ -\frac{7}{2000}(1-{{\rm{h}}}_{01})-\frac{{{\rm{e}}}^{3}}{10{(1+{{\rm{e}}}^{3})}^{2}}{h}_{01} & 0 & -0.07-\frac{1}{1+{{\rm{e}}}^{3}} & 0\\ \frac{0.01(1-{{\rm{n}}}_{01})}{{(1-{\rm{e}})}^{2}}+\frac{1}{640}{{\rm{n}}}_{01} & 0 & 0 & -\frac{1}{8}+\frac{0.1}{1-{\rm{e}}}\end{array}\right)$$

Based on the physiological parameters in section 3, Jacobian matrix of HH equation can be readily calculated as

11 $${\rm{A}}=\left(\begin{array}{cccc}-0.6773 & 69.1743 & 2.0474 & -55.1966\\ 0.0264 & -4.2236 & 0 & 0\\ -0.0041 & 0 & -0.1174 & 0\\ 0.0028 & 0 & 0 & -0.1832\end{array}\right)$$

Then, the eigenvalue of Jacobian matrix can be obtained as

12 $$\lambda =\left(\begin{array}{c}{\lambda }_{1}\\ {\lambda }_{2}\\ {\lambda }_{3}\\ {\lambda }_{4}\end{array}\right)=\left(\begin{array}{c}-4.6755\\ -0.2026+0.3824i\\ -0.2026-0.3824i\\ -0.1207\end{array}\right)$$

It is readily gained that ${R}_{e}(\lambda ) < 0$ takes place, which has all of negative real part. Thus, the derived system of HH model is asymptotic stability, which shows that the initial equation of HH model is validated to be asymptotic stability based on the indirect method of Lyapunov stability theory. Therefore the working equilibrium point of neuron has the performance of stability, which can provide some mechanism and behavior (a good performance of equilibrium point) for the bio-inspired electromagnetic protection.

## The Action Potential of Neuron System and Discussion

### The mathematic model of neuron system

From section 2, a simple neuron system is consisted of two neurons and one synapse. It is assumed that the electrical signal transfers from the preceding neuron to the posterior neuron, thus the action behavior of preceding neuron is not influenced by the synapse current. Based on HH model, the action potential of preceding neuron is calculated as

13 $$\left\{\begin{array}{c}\begin{array}{c}{C}_{pre}\frac{d{V}_{pre}}{dt}={G}_{Napre}{{m}_{pre}}^{3}{h}_{pre}({E}_{Napre}-{V}_{pre})+{G}_{Kpre}{{n}_{pre}}^{4}({E}_{Kpre}-{V}_{pre})+{G}_{Lpre}({E}_{Lpre}-{V}_{pre})+{I}_{pre}\end{array}\\ \frac{d{m}_{pre}}{dt}={\alpha }_{mpre}(1-{m}_{pre})-{\beta }_{mpre}{m}_{pre}\\ \frac{d{h}_{pre}}{dt}={\alpha }_{hpre}(1-{h}_{pre})-{\beta }_{hpre}{h}_{pre}\\ \frac{d{n}_{pre}}{dt}={\alpha }_{npre}(1-{n}_{pre})-{\beta }_{npre}{n}_{pre}\end{array}\right.$$

Moreover, due to the effect of synapse, the stimuli current of posterior neuron is sum of self-stimuli current I_B_ and transfer current of preceding neuron I_A_B_. Based on HH model, the action potential of posterior neuron is calculated as

14 $$\left\{\begin{array}{c}\begin{array}{cc}{C}_{post}\frac{d{V}_{post}}{dt}\,= & {G}_{Napost}{{m}_{post}}^{3}{h}_{post}({E}_{Napost}-{V}_{post})+{G}_{Kpost}{{n}_{post}}^{4}({E}_{Kpost}-{V}_{post})\\  & +\,{G}_{Lpost}({E}_{Lpost}-{V}_{post})+{I}_{post}+{I}_{A\_B}\end{array}\\ \frac{d{m}_{post}}{dt}={\alpha }_{mpost}(1-{m}_{post})-{\beta }_{mpost}{m}_{post}\\ \frac{d{h}_{post}}{dt}={\alpha }_{hpost}(1-{h}_{post})-{\beta }_{hpost}{h}_{post}\\ \frac{d{n}_{post}}{dt}={\alpha }_{npost}(1-{n}_{post})-{\beta }_{npost}{n}_{post}\end{array}\right.$$

Where C_pre_ indicates the membrane capacitance of preceding neuron, V_pre_ indicates the action potential of preceding neuron, gNa_pre_ indicates the sodium conductance of preceding neuron, gK_pre_ indicates the potassium conductance of preceding neuron, G_Napre_ indicates the maximum sodium conductance of preceding neuron, G_Kpre_ indicates the maximum potassium conductance of preceding neuron, G_Lpre_ indicates the maximum leak conductance of preceding neuron, E_Napre_ indicates the reversal sodium potential of preceding neuron, E_Kpre_ indicates the reversal potassium potential of preceding neuron, E_Lpre_ indicates the reversal leak potential of preceding neuron, m_pre_ indicates the activation parameter of sodium ion of preceding neuron, h_pre_ indicates the deactivation parameter of sodium ion of preceding neuron, n_pre_ indicates the activation parameter of potassium ion of preceding neuron, I_pre_ indicates the stimuli current of posterior neuron, V_restpre_ indicates the rest potential of preceding neuron, C_post_ indicates the membrane capacitance of posterior neuron, V_post_ indicates the action potential of posterior neuron, gNa_post_ indicates the sodium conductance of posterior neuron, gK_post_ indicates the potassium conductance of posterior neuron, G_Napost_ indicates the maximum sodium conductance of posterior neuron, G_Kpost_ indicates the maximum potassium conductance of posterior neuron, G_Lpost_ indicates the maximum leak conductance of posterior neuron, E_Napost_ indicates the reversal sodium potential of posterior neuron, E_Kpost_ indicates the reversal potassium potential of posterior neuron, E_Lpost_ indicates the reversal leak potential of posterior neuron, m_post_ indicates the activation parameter of sodium ion of posterior neuron, h_post_ indicates the deactivation parameter of sodium ion of posterior neuron, n_post_ indicates the activation parameter of potassium ion of posterior neuron, I_post_ indicates the stimuli current of posterior neuron, V_restpost_ indicates the rest potential of posterior neuron.

### The mathematic model of synapse

In order to simplify the analysis, *I*_*A_B*_ in Eq. (9) is assumed to be equal to the synapse current *I*_*syn*_, then

15 $${I}_{A\_B}={I}_{syn}$$

As is known to us, the synapse is consisted of chemical synapse and electrical synapse. The simplest mathematical model of chemical synapse^15^ is calculated as

16 $${I}_{syn}={G}_{syn}H({V}_{pre}(t-\tau )-{V}_{thresh})$$

Where G_syn_ indicates the strength of coupling, H indicates the step function of Heaviside, τ indicates the transfer delay time from the preceding neuron to posterior neuron, V_thresh_ indicates the threshold value of synapse.

A typical mathematical model of electrical synapse is calculated as

17 $${I}_{syn}={G}_{syn}({V}_{pre}(t-\tau )-{V}_{post})$$

Where G_syn_ indicates the strength of coupling, which is regard as the gap conductance.

Based on two kinds of synapse, a simple mathematical model of connect section (simplified synapse) we propose for the electromagnetic protection is calculated as

18 $${I}_{syn}={G}_{syn}({V}_{pre}(t)-{V}_{restpost})$$

Then, the synapse current is only dependent on the action potential of preceding neuron and a constant V_restpost_, which simplifies the Heaviside function in the chemical synapse and the action potential of posterior neuron V_post_ in the electrical synapse. The aim of simplified synapse we propose is to simplify the design of bio-inspired circuit.

### The numerical analysis and discussion

It is readily observed that the Eq. (14) has the performance of the strong coupling and nonlinear characteristics, which gives us some difficulties to solve it analytically. Thus, it needs to be calculated by way of numerical algorithm to obtain the action behavior. Because of a typical example of action potential among from action behavior, it is mainly focused on in this section. In order to simplify the analysis, the coupling strength (G_syn_) is assumed to be a constant value. The delay time τ is assumed to be equal to 0. The preceding neuron and posterior neuron are assumed to have the whole same biological parameters as follow:

19 $$\left\{\begin{array}{c}{{\rm{ccc}}}_{{\rm{pre}}}={{\rm{C}}}_{{\rm{post}}}={{\rm{C}}}_{1}\\ {{\rm{V}}}_{{\rm{restpre}}}{={\rm{V}}}_{{\rm{restpost}}}{={\rm{V}}}_{{\rm{rest1}}}\\ {{\rm{G}}}_{{\rm{Napre}}}{={\rm{G}}}_{{\rm{Napost}}}{={\rm{G}}}_{{\rm{Na1}}}\\ {{\rm{G}}}_{{\rm{Kpre}}}{={\rm{G}}}_{{\rm{Kpost}}}{={\rm{G}}}_{{\rm{K1}}}\\ {{\rm{G}}}_{{\rm{Lpre}}}{={\rm{G}}}_{{\rm{Lpost}}}{={\rm{G}}}_{{\rm{L1}}}\\ {{\rm{m}}}_{{\rm{0pre}}}{={\rm{m}}}_{{\rm{0post}}}{={\rm{m}}}_{01}\\ {{\rm{h}}}_{{\rm{0pre}}}{={\rm{h}}}_{{\rm{0post}}}{={\rm{h}}}_{01}\\ {{\rm{n}}}_{{\rm{0pre}}}{={\rm{n}}}_{{\rm{0post}}}{={\rm{n}}}_{01}\\ {{\rm{E}}}_{{\rm{Napre}}}{={\rm{E}}}_{{\rm{Napost}}}{={\rm{E}}}_{{\rm{Na1}}}\\ {{\rm{E}}}_{{\rm{Kpre}}}{={\rm{E}}}_{{\rm{Kpost}}}{={\rm{E}}}_{{\rm{K1}}}\\ {{\rm{E}}}_{{\rm{Lpre}}}{={\rm{E}}}_{{\rm{Lpost}}}{={\rm{E}}}_{{\rm{L1}}}\\ {{\rm{\alpha }}}_{{\rm{m0pre}}}{={\rm{\alpha }}}_{{\rm{m0post}}}{={\rm{\alpha }}}_{{\rm{m01}}}\\ {{\rm{\beta }}}_{{\rm{m0pre}}}{={\rm{\beta }}}_{{\rm{m0post}}}{={\rm{\beta }}}_{{\rm{m01}}}\\ {{\rm{\alpha }}}_{{\rm{h0pre}}}{={\rm{\alpha }}}_{{\rm{h0post}}}{={\rm{\alpha }}}_{{\rm{h01}}}\\ {{\rm{\beta }}}_{{\rm{h0pre}}}{={\rm{\beta }}}_{{\rm{h0post}}}{={\rm{\beta }}}_{{\rm{h01}}}\\ {{\rm{\alpha }}}_{{\rm{n0pre}}}{={\rm{\alpha }}}_{{\rm{n0post}}}={{\rm{\alpha }}}_{{\rm{n01}}}\\ {{\rm{\beta }}}_{{\rm{n0pre}}}={{\rm{\beta }}}_{{\rm{n0post}}}={{\rm{\beta }}}_{{\rm{n01}}}\\ {{\rm{V}}}_{{\rm{0pre}}}={{\rm{V}}}_{{\rm{0post}}}={{\rm{V}}}_{01}\\ {{\rm{m}}}_{{\rm{0pre}}}={{\rm{m}}}_{{\rm{0post}}}={{\rm{m}}}_{01}\\ {{\rm{h}}}_{{\rm{0pre}}}={{\rm{h}}}_{{\rm{0post}}}={{\rm{h}}}_{01}\\ {{\rm{n}}}_{{\rm{0pre}}}{={\rm{n}}}_{{\rm{0post}}}={{\rm{n}}}_{01}\end{array}\right.$$

**Figure 6:**
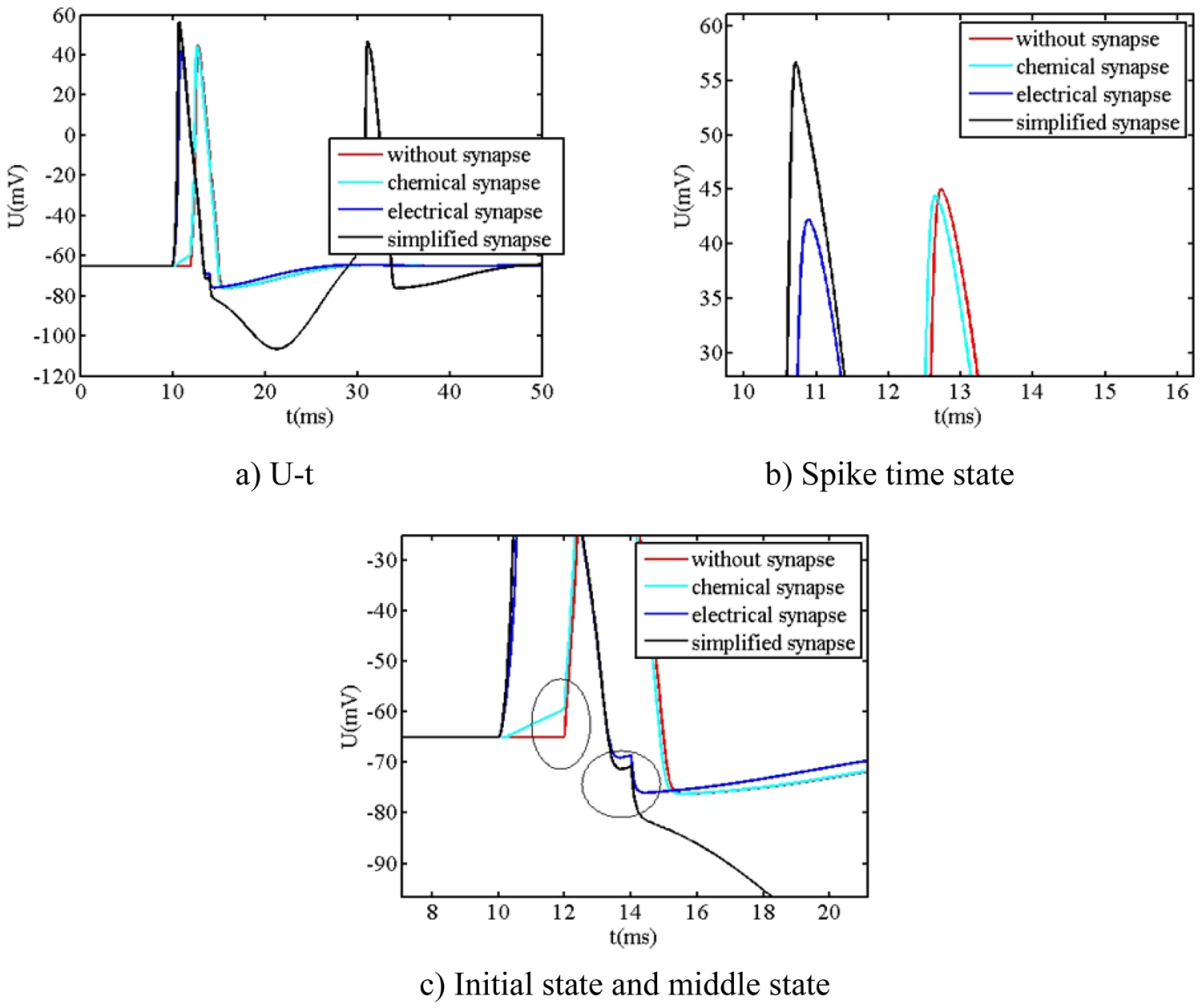
The relationship between action potential and different synapses.

The other simulated parameters of neuron system are the same to the ones of single neuron: C_1_ is equal to 1 μF/cm^2^, V_rest1_ is equal to −65 mV, G_syn_ is equal to 4, the values of simulated parameters of G_Na1_, G_K1_, G_L1_, E_Na1_, E_K1_, E_L1_, α_m01_, β_m01_, α_h01_, β_h01_, α_n01_, β_n01_, are obtained in section 3.2. The initial values of parameters are: V_01_ = −65 mV, m_01_ = 0.0529, h_01_ = 0.5961, n_01_ = 0.3177. The stimuli current intensities of preceding neuron and posterior neuron are both the single pulse signal: 100 μA/cm^2^ amplitude and 2 ms pulse width. The stimuli time of preceding neuron (t_sipre_) is equal to 10 ms while the stimuli time of posterior neuron (t_sipost_) is equal to 12 ms. Based on RK algorithm, the relationship between the action potential and different synapses is compared, as shown in Fig. 6.

From Fig. 6, it is readily observed that the action potential of posterior neuron with synapse is influenced by the different type of synapses, which all differs from the action potential of posterior neuron without synapse. When the preceding neuron and posterior neuron are connected by the different synapses, the action potential occurs to have the different dynamical responses: (1) The dynamical response has the similar behavior by way of chemical synapse and no synapse, which has the main difference that the initial state of chemical synapse has the small step (the left ellipse shown in figure c). (2) The dynamical response has the similar behavior by way of electrical synapse and simplified synapse, however, they have the big difference that the dynamical response of simplified synapse has two spikes, while the one of electrical synapse has only one spike, and the amplitude of first spike of simplified synapse (about 57 mV) is larger than the one of electrical synapse (about 42 mV). Additionally, the dynamical response of simplified synapse has a large decline, while the one of electrical synapse do not have the similar behavior rather than a small decline then repolarization (the right ellipse shown in figure c).

**Figure 7:**
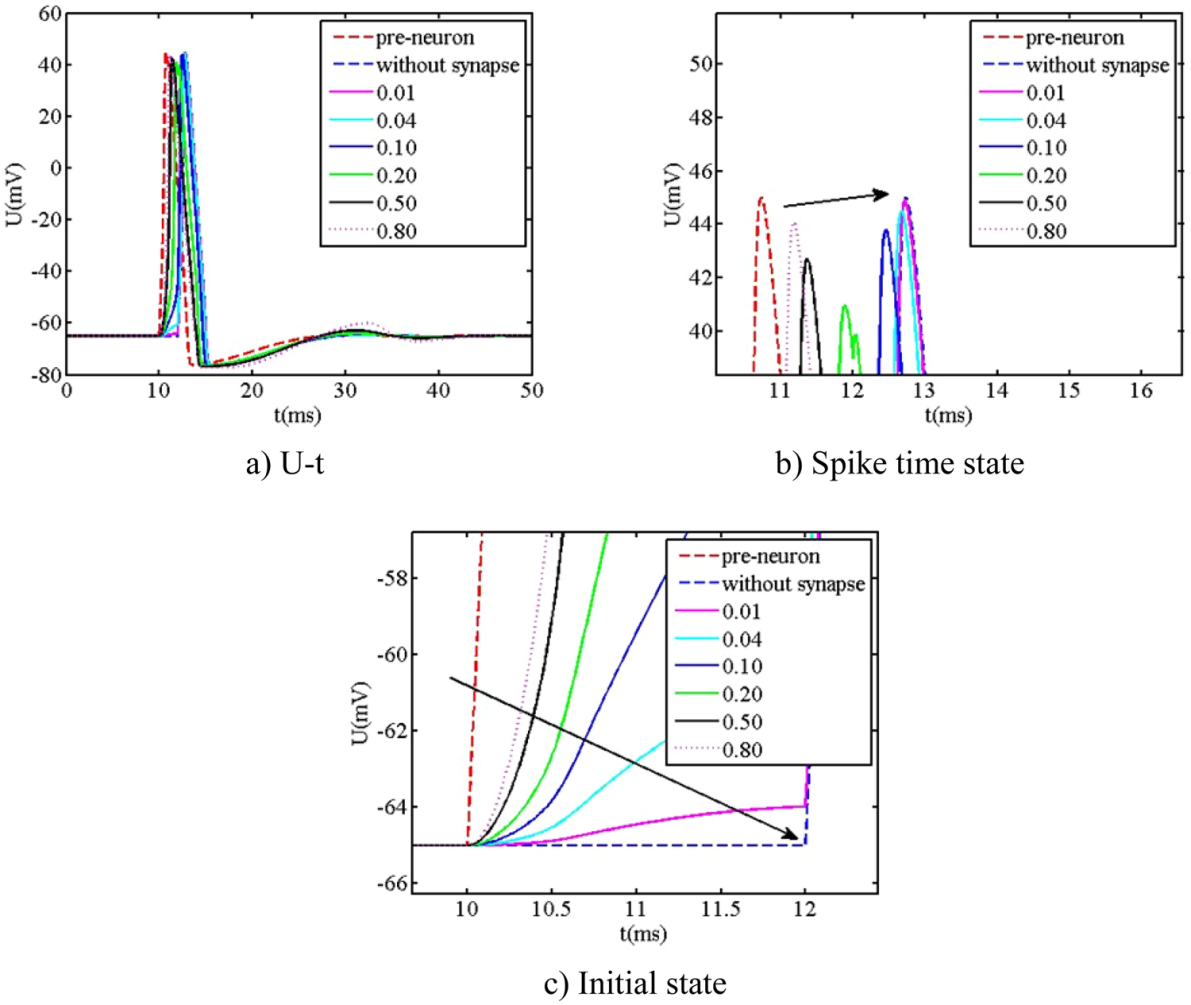
The relationship between action potential and connected weight.

In order to easily provide the foundational parameters for the design of bio-inspired circuit, the simplified synapse is mainly focused on. The comparison between the action potential and different coupling strength (the connected weight) of simplified synapse is shown in Fig. 7.

**Figure 8:**
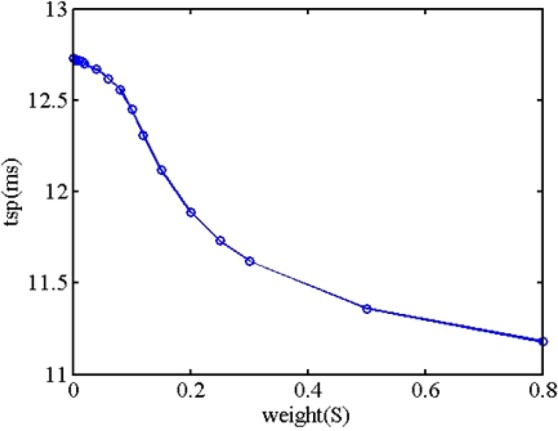
The spike time versus connected weight.

From Fig. 7, it is readily observed that the behavior of action potential is largely influenced by the connected weight. When the connected weight is equal to 0.2, two close spikes of action potential occur. When the connected weight is not less than 0.5, the remarkable wave at the time about 30 ms is observed. Additionally, it is clearly seen that the rise rate of initial state and spike time state is largely influenced by the connected weight, but the amplitude of action potential is less influenced by the connected weight. When the connected weight is smaller, the rise rate of initial state is closer towards the one of posterior neuron without synapse. When the connected weight is bigger, the rise rate of initial state is closer towards the one of preceding neuron. Furthermore, the spike time of action potential includes the information that the neuron carries out the physiology behavior. The spike time is different, and the physiology behavior may be different. Based on the above calculated results, the relationship between spike time and connected weight is extracted, as shown in Fig. 8.

**Figure 9:**
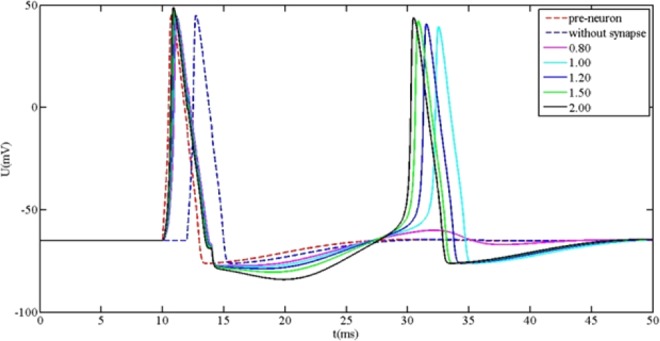
The relationship between action potential and lager connected weight.

From Fig. 8, it is clearly seen that the spike time slowly decreases then dramatically decreases then slowly decreases as the connected weight increases. The spike time is equal to 12.73 ms when the connected weight is equal to 0. At that time, the spike time of action potential of posterior neuron is only influenced by the self-stimuli current without the influence of preceding neuron. When the connected weight is equal to 0.8, the spike time is equal to 11.18 ms, which has the 12.2% relative error with respect to the spike time in state of no connected weight. When the connected weight is more than 0.8, the action potential of two spikes occurs, which is different from the case of one spike. The relationship between action potential of posterior neuron and connected weight (large value) is shown in Fig. 9.

From Fig. 9, two spikes are readily observed when the connected weight is not less than 1, which implies that the behavior of action potential can create the qualitative change by way of connected weight. One spike is at from 10 ms to 12 ms, while the other spike is at from 30 ms to 35 ms. The amplitudes of two spike are approximately close, which implies that the connected weight do not almost influence the amplitude. The spike time of the first spike is less influenced by the connect weight, while the spike time of the second spike is largely influenced by the connect weight. The phenomena imply that the action potential of posterior neuron has performance of abundant behavior information when the connected weight occurs to change, which can be applied to guide the design of bio-inspired circuit.

## Conclusion

Facing on the bio-inspired electromagnetic protection, the dynamical characteristics of neuron system consisted of two neurons and a synapse is investigated. The initial value problem of single neuron is analyzed, and the phenomenon is observed that when the initial value is not appropriate, some special abnormal action behaviors can happen, such as slow decrease, slow increase, and even an advanced sudden spike. The working stability of neuron is validated by way of Lyapunov stability theory, which can provide a good equilibrium point for the bio-inspired electromagnetic protection. The relationship between action potential of posterior neuron and different synapse are performed to be compared, which shows that the action potential (mainly spike time and initial state) varies largely when two neurons are connected with the different synapses. The simplified synapse we propose for the electromagnetic protection is mainly focused on. The spike time is observed to decrease as the connected weight increases and the amplitude is observed to be not almost influenced by the connected weight. The phenomenon of two spikes is observed when the connected weight is not less than 1. These simulated results will provide some datum for the design of bio-inspired circuit.
